# Impact of the Different Preparation Methods to Obtain Human Adipose-Derived Stromal Vascular Fraction Cells (AD-SVFs) and Human Adipose-Derived Mesenchymal Stem Cells (AD-MSCs): Enzymatic Digestion Versus Mechanical Centrifugation

**DOI:** 10.3390/ijms20215471

**Published:** 2019-11-02

**Authors:** Pietro Gentile, Claudio Calabrese, Barbara De Angelis, Jacopo Pizzicannella, Ashutosh Kothari, Simone Garcovich

**Affiliations:** 1 Surgical Science Department, Plastic and Reconstructive Surgery, University of Rome “Tor Vergata”, 00179 Rome, Italy; bdeangelisdoc@gmail.com; 2 Breast Unit Department, San Rossore Clinic, 56122 Pisa, Italy; claudiocalabrese.it@gmail.com; 3 ASL02 Lanciano-Vasto Chieti, Ss. Annunziata Hospital, 66100 Chieti, Italy; jacopo.pizzicannella@unich.it; 4 Chief of Breast Surgery Unit, Guy’s Hospital, Guy’s and St. Thomas’ NHS Foundation Trust, London SE1 9RT, UK; Ashutosh.Kothari@gstt.nhs.uk; 5 Institute of Dermatology, F. Policlinico Gemelli IRCSS, Università Cattolica del Sacro Cuore, 00168 Rome, Italy; simgarko@yahoo.it

**Keywords:** stromal vascular fraction cells, SVFs, adipose-derived mesenchymal stem cells, AD-MSCs, stem cell isolation, kit of AD-SVFs isolation, kit fat graft, regenerative plastic surgery

## Abstract

Autologous therapies using adipose-derived stromal vascular fraction (AD-SVFs) and adult adipose-derived mesenchymal stem cells (AD-MSCs) warrant careful preparation of the harvested adipose tissue. Currently, no standardized technique for this preparation exists. Processing quantitative standards (PQSs) define manufacturing quantitative variables (such as time, volume, and pressure). Processing qualitative standards (PQLSs) define the quality of the materials and methods in manufacturing. The purpose of the review was to use PQSs and PQLSs to report the in vivo and in vitro results obtained by different processing kits that use different procedures (enzymatic vs. non-enzymatic) to isolate human AD-SVFs/AD-MSCs. PQSs included the volume of fat tissue harvested and reagents used, the time/gravity of centrifugation, and the time, temperature, and tilt level/speed of incubation and/or centrifugation. PQLSs included the use of a collagenase, a processing time of 30 min, kit weight, transparency of the kit components, the maintenance of a closed sterile processing environment, and the use of a small centrifuge and incubating rocker. Using a kit with the PQSs and PQLSs described in this study enables the isolation of AD-MSCs that meet the consensus quality criteria. As the discovery of new critical quality attributes (CQAs) of AD-MSCs evolve with respect to purity and potency, adjustments to these benchmark PQSs and PQLs will hopefully isolate AD-MSCs of various CQAs with greater reproducibility, quality, and safety. Confirmatory studies will no doubt need to be completed.

## 1. Introduction

Surgical procedures using autologous cell based approaches that include harvesting, minimal manipulation, and immediate transplant as a one-step process open the doors to tremendous possibilities in present day regenerative surgery. Systems that isolate adult adipose-derived mesenchymal stem cells (AD-MSCs) have found clinical application in different fields. These procedures require the collection and preparation of fat tissue (FT) to obtain the desired end products, namely, adipose-derived stromal vascular fraction (AD-SVFs) and AD-MSCs. Such procedures make the patient’s own cells available through safe methods and do not lead to adverse reactions. Many other important cell types could be harvested through these minimally invasive strategies [1]. The process bypasses a large number of limitations linked to exogenous cell treatments. This is made possible by avoiding in vitro cell control and expansion, which requires good manufacturing practice (GMP) approved facilities. In addition, they could prove beneficial in terms of maintaining a strategic distance from cell culture by limiting changes in phenotype, consequent to the removal of cells from their local microenvironment [1].

Additionally, these procedures can be performed within a surgical space, where growth by cell culture is not necessary or not performed, reducing the time for the procedure itself. The EMA (i.e., European Medicines Agency), FDA (i.e., US Food and Drug Administration), and other similar bodies view adult cells as biological products that can be split into two classes. The first class has undergone a small amount of manipulation through processes such as filtration, centrifugation, isolation, and more. The second are those that have undergone a significant manipulation process such as those found in stem cells that have been expanded through cell cultures. Some intraoperative techniques could easily fall into the category of minimal manipulation of cell products. Broad clinical trials are typically not required for this set, and leads to the speeding up of possible interpretations for facilities.

The processing of tissues in the operating room such as in the preparation of FT to be used in autologous fat grafting requires a level of surgical skill and judgment to not only maintain standard operating procedures (SOPs), but also to optimize the intended result. Accordingly, human skill and judgment must account for a seemingly countless number of patient and surgeon variables for surgical success. In this case, the SOPs of the surgical techniques help to limit the variables to more reliably determine the quality of the surgical outcomes.

The field of regenerative plastic surgery relies on the preparation of adipose tissue and the isolation of cells to be used for patient treatments instead of pharmaceuticals. 

Isolation systems used in regenerative plastic surgery each have their own SOPs that may comply with current good manufacturing practices (C-GMPs). However, these systems are not configured for specific protocols. C-GMPs are regulations outlined by the FDA which “provide for systems that assure proper design, monitoring, and control of manufacturing process and facilities which assures identity, strength, quality, and purity of drug products by requiring that manufacturers of medications adequately control manufacturing operations” (https://www.fda.gov/drugs/developmentapprovalprocess/manufacturing/ucm169105.htm).

Similar to post-operative quality metrics that judge surgical outcomes, so also are the tissues and cells isolated for use in regenerative plastic surgery judged for quality. The characteristics used to define the quality of prepared tissues and isolated cells are collectively known as critical quality attributes (CQAs). According to the FDA, a CQA is a “physical, chemical, biological, or microbiological property or characteristic that should be within an appropriate limit, range, or distribution to ensure the desired product quality.” (FDA, Guidance for industry. Q8(R2) pharmaceutical development. 2009; November (Revision 2)).

The FDA expects manufacturers to “identify critical parameters in the manufacturing process and critical product attributes to ensure the desired clinical effect of the final product” (https://www.fda.gov/downloads/biologicsbloodvaccines/guidancecomplianceregulatoryinformation/guidances/xenotransplantation/ucm092705.pdf).

CQAs of clinical grade therapeutic products include cellular safety, identity, purity, and potency (FDA. Guidance for industry. Q8 (R2) pharmaceutical development. 2009; November (Revision 2)).

Processing quantitative standards (PQSs) and processing qualitative standards (PQLSs) may help to manufacture or prepare tissues and cells with specific attributes.

PQSs are metrics used to standardize manufacturing quantitative variables such as time, volume, and pressure. PQLSs are metrics used to standardize the quality of the materials and methods of manufacturing. PQSs and PQLSs help determine the quantity and quality of manufactured products while improving manufacturing reproducibility.

The objective of this review was to compare a collagenase-based procedure with a minimal-manipulation based technique using centrifugation and filtration by using PQSs and PQLSs to isolate AD-SVFs and their AD-MSC subpopulations from freshly harvested FT. To conform with the manufacturing PQSs and PQLSs, both these procedures employ a closed preparation system (the “System”) in the form of a kit (the “Kit”). The preparation system includes a kit as well as ancillary devices that include a small centrifuge and incubating rocker. Targeted PQSs included the volumes of lipoaspirate and reagents, the time and gravity of centrifugation, and the time, temperature, tilt level, and tilt speed of incubation. Targeted PQLSs included the use of a CGMP collagenase, a processing time of 30 min or less, kit content weight of 2 lbs. or less, the maintenance of a continuously closed sterile processing environment, and the use of a small centrifuge and incubating rocker configured by the manufacturers for use with the kit.

## 2. European and FDA Regulations Regarding the Application of AD-SVFs and AD-MSCs

The EMA and FDA consider cell products as biological entities that are split into two sets. The first consists of those with minimal manipulation and the second are those that are significantly manipulated. Law number 1394/2007 of the European Parliament (EP) for cutting-edge procedures defines “bio-process engineering products”, which sets aside anything containing or specifically made of cells and tissues, either human or animal, that is non-vital in nature and has no immunologic, pharmacologic, or metabolic activity. Included amongst the advanced therapy pharmaceutical products are ones used for gene and somatic cell treatment (Directive 2001/83/EP, Annex I). Cells and tissues are to be viewed as products of the bio-process engineering in the event that they” experience “extensive manipulation”. 

The rule itself is a contrast between the two types of manipulation under discussion. Manipulations that are typically not seen as having undergone bio-process engineering will typically include forming, granulating, cutting, purification, centrifugation, nitrification, absorption of antimicrobial or antitoxin arrangements, cleansing, partition, and lyophilization. 

“The definition of drugs for advanced therapy excludes non-repetitive preparations finished as per doctor’s supervision, in terms of them implementing a single remedy for a product meant for a particular patient, whereby they do not disregard important standards pertaining to security and quality. With the introduction of Article 17 of Law number 1394/2007 (the Advanced Therapy Medicinal Products (ATMPs) Regulation), applicants are required to approach the Committee for Advanced Therapies (CAT) with a proposal for the arrangement of ATMPs. 

ATMP characterizations rely on the evaluation of how the product justifies the different elements of GTMP (i.e., gene therapy medicinal products), TEPs (i.e., tissue-engineered products), and sCTPMPs (i.e., somatic cell therapy medicinal products). The discussion also looks at whether the product can live up to a consolidated ATMP’s definition. Furthermore, it is acknowledged that given the dynamic nature of the products in question, the limited information bundle at the start of the improvement process, alongside the speedy evolution of innovation and science, could lead to off-the-fringe inquiries. 

EMA/CAT Suggestions on Minimal Manipulation

In agreement with the reflection paper EMA/CAT/600280/2010 Rev 1, 20 June 2014, by the CAT, Line 10, according to which “a similar basic capacity for a cell populace implies that the cells, when expelled from their unique condition in the human body are used to maintain the original capacity in a similar anatomical or histological condition”, it is possible to presume that autologous use in a one-step medical procedure, minimal manipulation, omo-functional application “used for an indistinguishable fundamental capacity in the beneficiary as in the donor,”, and manipulation performed through devices in aseptic conditions (operatory room) would be conditions that do not require the rule’s application of good manufacturing practice (GMP) for preparation, good clinical practices (GCP) for clinical use, or ethical committee underwriting.

## 3. Cell Identity

AD-SVFs and AD-MSCs were documented by the International Federation for Adipose Therapeutics and Science (IFATS) in combination with the International Society for Cellular Therapy (ISCT) for the purpose of providing a standard that various research groups could follow. The two societies developed a document of identification for both AD-MSCs and AD-SVFs, where the literature was summarized so that it could be altered when new information, results, and data come to light through ongoing clinical work. As per the declaration, cells needed a viability of ≥ 70% and ≥ 90% for AD-SVF and AD-MSC, respectively. Percentage of stromal progenitors evaluated with a fibroblast colony forming unit assay (CFU-F) is expected to be ≥1% for AD-SVFs and ≥5% for AD-MSCs. The AD-SVFs’ identity, phenotype, and functional characteristics of adipose-derived cellular fractions were considered closely related to the adipose microenvironment and identified through a typical marker profile. In detail, the AD-SVFs immunophenotype should display the following typical marker profile for stromal cells: CD44, CD73, CD13, CD90, CD29 positive (≥40%), and CD34 positive (≥20%), but CD31 (≤20%), and CD45 negative (≤50%). In contrast, AD-MSCs should be positive for CD29, CD13, CD44, CD90, CD73, and CD105 (>80%), but negative for CD31, CD45, and CD235a (≤2%). In addition, AD-MSCs are expected to have the potentiality to differentiate into the osteogenic, chondrogenic, and adipogenic lineage. The differentiation capacity can be evaluated by histological staining like Alizarin red or von Kossa for osteogenic differentiation, Alcian Blue, or Safranin O for chondrogenic differentiation and Oil red O or Nile red for adipogenic differentiation [2].

## 4. Methods of AD-SVF Isolation 

### 4.1. Enzymatic Versus Non-Enzymatic Digestion of Fat Tissue to Obtain AD-SVFs

Enzymatic digestion of FT is the most used isolation method to obtain AD-SVFs. “Typically, collagenase, dispase, or trypsin are used for the digestion of FT [2]. Even though the techniques for the isolation of said cells are fairly diverse, they still stick to a standard process. The lack of proper quantification has to do with the different of steps of the washing, centrifugation parameters, enzyme concentration levels, erythrocyte lysis techniques alongside filtration, and finally, the conditions that the cultures are subjected to [3,4,5,6,7,8,9,10,11]. If the tissue in question is washed and subsequently put through enzymatic digestion, the cells that are split through centrifugation from mature adipocytes produce enzymes and oil solution. “Frequently used collagenases are type I and type II [8,11,12]. GMP degree collagenases are produced through recombinant bacteria and typically are provided in lyophilized form. Enzyme concentrations, expressed as weight per volume percent (*w*/*v*), have been reported in the literature with a range from 0.075% (*w*/*v*) to 0.3% (*w*/*v*) [7]. An erythrocyte lysis phase was commonly included to get rid of erythrocyte contamination. Performing an additional washing phase, the AD-SVFs suspension was either cultured or cryopreserved in expansion medium. The plastic-adherent cell fraction including AD-MSCs can be obtained after passaging or cryopreservation, or further cultivated for expansion for a more homogeneous AD-MSC population. As the application of enzymes is characterized by high costs and might have an impact on safety [5] and efficacy [13,14], several physicians and researchers have focused on non-enzymatic procedures using centrifugation, filtration, and micro-fragmentation. Mechanical procedures like centrifugation, filtration, and micro-fragmentation replaced the enzymatic digestion to isolate AD-SVFs and AD-MSCs. However, similar to the enzymatic procedures, the range of protocols for the non-enzymatic procedures also showed high variations.

### 4.2. Procedures, Kit, and Systems Based on Enzymatic Digestion

Aesthetic surgery-based experiences have shown that transferring autologous fat is helpful and will aid in short-acting fillers [15,16]. However, their use can lead to significant problems including necrosis and fat graft resorption. In order to augment the retained percentage of fat grafts reducing the percentage of necrosis, angiogenesis is needed for nutrition and incorporation within the surrounding tissue. Further implementation of AD-SVFs and AD-MSCs can get rid of issues such as necrosis and fat resorption through an improvement of angiogenesis. Cell-assisted lipotransfer (CAL) can lead to a fall in postoperative atrophy and augment neovascularization [17,18,19,20,21,22,23]. Automatic devices can aid in standardizing the isolation process by ensuring that the process is sterile. In effect, many firms and research labs have begun creating automatic equipment to take up a part, or in some cases, the entire cell isolation. Some of the pieces of equipment were created to enrich FT for autologous grafts during regenerative plastic surgery and later improved for the isolation of cell fractions.

The following kits and systems focus on automatic AD-SVF isolation by collagenase based digestion: Celution^®^ 800-CRS and 820-CRS (Cytori Therapeutics-Inc., San Diego, USA); STEM-X™ (Medikan International-Inc., Seul, Korea); Beauty Cell (N-Biotek-Inc., South Korea); GID SVF-1™ (GID Group-Inc., Louisville, KY, USA); HuriCell (Hurim-BioCell, Co. Ltd., South Korea); AdiStem™ Small/Large Kit and AdiLight (AdiStem-Pty Ltd., China); Sepax 2 (Biosafe-Group SA, Switzerland); Cellthera Kit I and II and Method for isolation of SVFs (Cellthera s.r.o., Czech Republic); A-Stromal™ kit (Cellular-Bio-medicine Group Inc., Cellular-Biomedicine Group HK, Ltd., New York, USA); Sceldis^®^ (ED-Co. Ltd. and Purebiotech Co. Ltd., South Korea, Medica Group, United Arab Emirates); automated systems and methods for isolating regenerative cells from FT (General Electric-Company, Boston, MA, USA); apparatus and methods for cells isolation (Ingeneron-Inc., Houston, TX, USA); UNISTATION™ (Neo-Genesis-Co. Ltd., South Korea), CHA STATION™ and Multi Station (PNC-International Co. Ltd., South Korea-PNC); CID300 (SNJ-Co. Ltd., South Korea-TOPMED-CO. LTD., South Korea); Stempeutron™ (Stempeutics-Research Pvt. Ltd., Indiaclos Tissue Genesis Icellator Cell Isolation System) and Hand-held micro-liposuction adipose harvester, processor, and cell concentrator (Tissue Genesis-Inc., Honolulu, USA).

### 4.3. Procedures, Kit, and Systems Based on Non-Enzymatic Digestion

An alternative to collagenases and enzymatic digestion is represented by mechanical procedures based on non-enzymatic digestion like centrifugation and filtration. Mechanical kits and systems commonly used for harvesting and purifying FT containing endothelial cells include: Fastkit (CORIOS-Soc.-Coop., Italy); LipiVage™ (Genesis-Biosystems-Inc., Lewisville, TX, USA); Lipogems^®^ (Lipogems-International-S.p.A., Italy); Lipo-Kit GT (Medikan-International-Inc., Seul, Korea); StromaCell™ (Micro-Aire-Surgical Instruments, LLC, Charlottesville, VA, USA); MyStem^®^ (MyStem-LLC, United States); and Revolve™/GID 700™ (Life-Cell Corporation, United States, GID Group-Inc., Branchburg, NJ, USA). 

Several other non-enzymatic kits and/or procedures aimed at the isolation of ASCs and to obtain SVFs are reported below: Method to obtain AD-SVFs (Agency-Science, Tech & Res, China); procedure and device for separating adult stem cells from fatty tissue and device for separating adult stem cells (Human-Med AG, Germany); ultrasonic cavitation derived stromal or mesenchymal vascular extracts and cells derived there from obtained from FT and use thereof and isolation of AD-SVFs (Intelli-Cell-BioSciences-Inc., New Yor, NY, USA); non-enzymatic method for isolating human AD-MSCs (Pennington-Biomedical-Research Center, Baton Rouge, LA, USA); isolation of stem cells from FT by ultrasonic cavitation and methods of use (Rusty-Property-Holdings Pty Ltd., Australia, Amberdale-Enterprises Pty Ltd.,); and selective lysing of cells using ultrasound (Solta-Medical, Inc., Hayward, CA, USA).

Due to evolution in the market, some of the kits/devices/society names might have modified.

## 5. In Vitro/In Vivo Evaluation of AD-SVFs 

### 5.1. In Vitro/In Vivo Evaluation of AD-SVFs and AD-MSCs Obtained from Devices/Procedures Based on Enzymatic Digestion

Many of the kits, devices, and procedures reported have undergone testing in both pre-clinical and clinical research settings. Moreover, additional papers have also focused on implementing different systems to isolate cells. Some of these have reported that AD-SVFs were isolated with the AdiStem™ cell isolation kit in combination with PRP (i.e., platelet-rich plasma) and then added to NOD/SCID nude mice, producing joint regeneration [24]. In this case, the cells obtained by AdiStem™ were activated with AdiLight (photo-bio-stimulator) with a total viable cell number of 12 Å~ 10^6^/^mL^ fat compared to a standard isolation procedure with 10 Å~10^6^ cells/mL fat [25]. AD-SVFs activated with this procedure were endobronchially infused in patients affected by idiopathic pulmonary fibrosis (IPF), obtaining a marginal improvement of walking and forced vital capacity [26]. Another study on this procedure used AD-SVF infusion in IPF patients with no deterioration in functional parameters and quality of life [27]. An important paper was published by Michalek et al. [28] where 1128 patients affected by osteoarthritis were treated by injecting AD-SVFs, obtained with Cellthera kits, into the joint, showing pain and movement improvements. 

Enzymatic digestion of FT with the Celution^®^ 800/CRS system exhibited a cell number of 2.95 × 10^5^ cells/mL with a viability of 86.6% [29]. In a study where different kit and systems were compared, the Celution^®^ 800-CRS system demonstrated the highest cell yield (2.41 × 10^5^ cells/g) when compared to Multi-Station (1.07 × 10^5^ cells/g), Lipo-Kit GT (0.35 × 10^5^ cells/g), and CHA STATION™ (0.05 × 10^5^ cells/g) [30]. SVFs-enhanced fat graft obtained by the Celution^®^ system resulted in a reduction of fat resorption, increasing the angiogenesis in nude mice [31]. Autologous AD-SVFs isolated with the same procedure improved hand disability by reducing pain in systemic sclerosis [32] and when transurethrally infiltrated, resulted in a reduction of male stress urinary incontinence [33]. AD-SVF-enhanced fat graft injections with the Celution^®^ system improved breast contour in a clinical trial for breast conservation therapy (BCT) [34]. SVFs obtained by GID SVF-1™ were 7.19 ± 2.11 × 10^5^ nucleated cells/mL of dry FT. The cells number was influenced by the patient’s age, decreasing with increasing age [35]. AD-SVFs derived by the Sceldis^®^ device were mixed with PRP, hyaluronic acid, and CaCl^2+^ and used to treat knee pain due to meniscus tear [36,37]. The method of Khan et al. resulted in a ~6 × 10^5^ cells/mL (66% viability) for donor 1 and ~1 × 10^6^ cells/mL (51% viability) for donor 2 [38]. Cells obtained by the HuriCell device were used in a pre-clinical model of focal cerebral ischemia, displaying neuro-protective effects [39]. Stubbers and Coleman [40] created a procedure for cell isolation yielding 4.9 × 10^6^–24.7 × 10^6^ total nucleated cells/100 g fat harvested. AD-SVF-enhanced fat graft, obtained by the Tissue Genesis Icellator Cell Isolation system, were used for face and breast augmentation or reconstruction [41]. In another study aimed to isolate AD-SVFs, Domenis et al. [42] analyzed three different kits (Lipo-Kit, Celution^®^, and Fastem). In this study, AD-SVF-enhanced fat grafts obtained with these procedures increased thickness and long-term maintenance of fat grafts in patients treated for outcomes of breast reconstruction when compared to not-enhanced fat grafting. However, the Fastem kit, based on the centrifugation and mechanical filtration of fat collected, was less effective in stem cell enrichment when compared to the enzymatic devices Lipo-Kit and Celution^®^.

### 5.2. In Vitro/In Vivo Evaluation of AD-SVFs and AD-MSCs Obtained from Devices/Procedures Based on non- Enzymatic Digestion

Filtration, washing, and purification of FT adopting Puregraft^®^ displayed augmented tissue viability and reduced quantity of red blood cells, free lipids, and contaminants when compared to other fat grafts [43,44]. FT washed and purified through GID 700™ exhibited significantly reduced amounts of lactate dehydrogenase, triglycerides, and hematocrit maintaining the adipose graft osmolarity [45]. AD-SVFs obtained by the harvesting and filtration device LipiVage™ displayed endothelial and mesenchymal progenitor cells, maintaining their differentiation capacity when used as fibrin spray [46]. LipiVage™ produced a higher number of adipocytes and sustained a higher level of intracellular enzyme (glycerol-3-phophatase dehydrogenase (G3PDH)) activity within fat grafts [47]. A study by Bianchi et al. [48] reported that cells obtained from Lipogems^®^ displayed a significantly higher concentration of mature pericytes, AD-MSCs, exosomes, and a lower quantity of hematopoietic cells when compared to isolated cells with enzymatic digestion [49]. In addition, this system showed paracrine and arteriogenic functions for the rescue of ischemic limb [50].

Compared to AD-MSCs isolated with enzymatic digestion (Table 1), the cells obtained by this procedure improved efficient direct multi-lineage reprogramming in human skin fibroblasts when exposed to a radio electric asymmetric conveyer (REAC) [51]. FT collected and micro-fragmented with this system produced a better mesenchymal stem cell amount when compared to fat harvested and not processed without modification of the structural composition of the same fat [52]. In orthognatic surgery, it reduced post-operative pain and swelling, improving the aesthetic results [53]; it improved the osteointegration, stability and healing of the implants in newly formed bone [54] and improved fecal incontinence symptoms [55]. 

An interesting system for harvesting and homogenizing FT for endothelial cells was described by Hu et al. [56] obtaining 1.12–2.13 Å~106 cells larger than 7.8 μm from 1 g of FT after enzymatic isolation of the non-enzymatic isolated cells. A great number of micro-vascular endothelial cells may be harvested using a cannula with cutting-edges to disrupt the connective tissue. Victor created a procedure that aimed to isolate AD-SVFs by applying ultrasonic cavitation and yielding 1.67–2.24 Å~107 cells with a viability of 97.1–98.9% [57]. Bright et al. [58] promoted the dissociation of FT by lysing mature adipocytes by applying ultrasonic cavitation with a yield of about 2–4 million cells/1 g fat. Many studies [36,37] have concluded in favor of intra-articular AD-SVF infiltration in people affected by osteoarthritis (knee, hip), with intra-venous AD-SVFs favored for rheumatoid osteoarthritis; the latter promoting improvement in pain, stiffness, and physical function. People affected by chronic migraine experienced a reduction in frequency and severity of migraines after systemic infusion of AD-SVFs obtained by the ultrasonic cavitation protocol [59]. Schafer [60], focused on the possibility of isolating fat derived cells using ultrasonic energy/acoustic standing waves, ultimately producing small (<50 μm), but more vital pre-adipocytes and stimulating angiogenesis. Gimble et al. [61] suggested an interesting and simple procedure based on the shaking and washing of fat and related AD-MSCs and AD-SVFs. Their process yielded 2.5 Å~106 cells per 100 mL fat, and maintained a high osteogenic and adipogenic differentiation capacity as compared to a classic enzymatic digestion procedure.

## 6. AD-SVFs, AD-MSCs, and Cancer Relationship 

AD-MSCs were in use long before their characterization as part of the fat grafting technique first described by Coleman in 1997 [63]. In fact, the injection of AD-SVFs-enhanced or AD-SVFs-not-enhanced fat injection is the most common procedure in post-oncologic breast reconstruction; with 62% of a surveyed 2584 American surgeons declaring that they regularly use fat grafting for this specific purpose [64]. In the last few years, AD-MSC-oriented solutions have been trialed and implemented in various clinical setups, oncological and otherwise, including chronic ischemic cardiomyopathy, inflammatory bowel disease, reconstruction for sarcoma of the soft tissues, rheumatoid arthritis, graft versus host disease, and others [65,66,67,68].

Alongside the potential of AD-MSCs as a therapeutic agent, there exists the possibility that they could possess a pro-oncogenic risk. A good amount of evidence now highlights obesity as a serious oncological risk factor; moreover, peri-tumor adipose tissue and its related progenitor cells are also seen as risk factors [69]. “Even though in many clinical studies AD-MSC has not been linked to an augmented risk of loco-regional or far cancer recurrence, there is no conclusive evidence or consensus medical opinion with regard to its safety yet. This notwithstanding, the link between it and cancer has been investigated thoroughly through preclinical models [70]. 

Furthermore, they may perform a function in promoting the growth of tumors, metastatic potential, and invasiveness through different pathways. Their proangiogenic function though the release of chemokines and growth factors such as PDGF, VEGF, and c-kit promote endothelial cell proliferation and a cancer-supporting vascular network development [71,72,73]. Moreover, AD-MSCs have immunomodulating proprieties mediated by TGF-β1, HGF, IDO, and INF-γ that impair immune-mediated responses to tumor [73,74,75,76,77,78]. They also have the capacity to induce cell proliferation in the cell line MCF-7/ADR (a multidrug-resistant breast cancer cell model) and drug resistance through C-terminal Src kinase (Csk)-binding protein (Cbp) expression [79].

The transition from epithelial to mesenchymal (EMT) has been reported as a fundamental passage in cancer growth and its evolution toward a metastatic and more invasive phenotype [80]. To this end, AD-MSCs may promote EMT in cells of breast cancer (BC) through several pathways, particularly acting through PI3K/AKT signaling and p38 MAP kinase [81,82], or alternatively by overexpressing leptin, as identified by AD-MSCs coming from patients affected by obesity [83]. Additionally, the inhibition of Wnt signaling into cancer-associated fibroblasts has been reported to produce AD-MSCs transformation by BC-derived factors [84,85]. Myofibroblastic differentiation was also observed in AD-MSCs exposed to exosomes of BC for the induction of TGF-β signaling, [85] and AD-MSC are themselves able to secrete exosomes, inducing breast cancer cells (BCCs) migration mediated by Wnt-signaling [86]. 

The involvement of AD-MSCs into the neoplastic microenvironment has been regarded as controversial, as it is not limited to near cells. This phenomenon may be explained by the ability of tumor cells, its stroma, and inflammatory cells to release agents such as SDF-1 and MCP-1, which stimulate AD-MSC homing and migration to a cancer micro-environment [87].

A higher quantity of circulating AD-MSCs was identified in people with obesity affected by colo-rectal tumor, prostate tumor, and BC [88,89,90]. 

In another context, MSCs can be utilized as a Trojan Horse, exploiting their tumor-homing facets to deliver therapeutic anti-neoplastic agents directly to the tumor environment. AD-MSCs have undergone testing as vectors for many new solutions to cancer including micro-RNAs, drug-loaded nanoparticles, viral vectors, and others [91].

AD-MSCs present several benefits in comparison to other MSCs. Their harvesting is not invasive, and can produce a cell yield that is more than 1000-fold higher than cord-blood MSCs and bone-marrow MSCs [92,93]. In addition, they have demonstrated a higher proliferative capacity, longer life-span, and shorter doubling time and in vitro senescence when compared to bone-marrow MSCs [94]. This MSC–cancer interplay is characteristic of AD-MSCs, which has gained notoriety as a “double edged sword” in the contemporary medical community, in light of its various positive and negative aspects [95,96]. 

## 7. Adipose Microenvironment, Obesity Relationship, and Breast Cancer Modulation

As introduced previously, higher quantities of circulating AD-MSCs were identified in people affected by obesity [88,89,90]. Obesity is related with the growth of many cancer types like BC [97]. Adipocytes, a major cellular component in FT with a crucial role in maintaining the energy balance, are dysfunctional in people with obesity, promoting BC growth [97]. Dysfunctional adipocytes may secrete metabolic substrates as cytokines and adipokines that boost proliferation, progression, invasion, and migration of BCCs, altering gene expression profiles, inducing inflammation, and hypoxia inhibiting apoptosis. Additionally, it is known that excessive cholesterol, free fatty acids (FFAs), hormones, triglycerides, interleukins, leptin, and chemokines may boost the development of BC [97]. 

Accumulated fatty acids are absorbed directly by the cancers and create power for their growth through β-oxidation, representing the biggest source of ATP in the cancer. FFAs promote BC growth activating the EGF receptor, GTP-binding protein, and protein kinase C pathway [98], controlling cell proliferation via phosphatidylinositol 3-kinase (PI3K) [99], cell migration through FFAs receptor 1 and 4, and the activation of AKT pathway [100].

In people with obesity, a higher amount of cholesterol is detectable. However, the role of total cholesterol (TC) or high-density lipoprotein (HDL) and low-density lipoprotein (LDL) in BC development is not clear. A meta-analysis has displayed an inverse relationship between TC and HDL to the risk of BC development [101]. Both LDL and very-low-density lipoprotein (VLDL) exposure stimulates the development, migration, and invasion of BCCs through activation of the PIK3/AKT pathways, especially VLDL, which stimulate lung metastasis and cancer cells angiogenic activity [102].

Additionally, adipocyte exosomes contain proteins connected to fatty acid oxidation (FAO) [103]. Obesity boost exosomes that modulate FAO, thereby contributing to cancer diffusion [103]. Supplementary studies have also displayed that microRNAs released from adipose exosomes may promote BC growth and related invasion capability [104]. A total of 98 miRNAs secreted by adipose exosomes has been identified and among of these, miR-3184-5p seems to be the most upregulated, whereas miR-181c-3p seems to be the most downregulated for BC. Both focus on PPARα and FOXP4 [104]. Eight miRNAs are associated with BMI level and miR-191-5p, and miR-17-5p has been identified as involved in cancer development. In a detailed view, miR-191-5p upregulated 17β-estradiol protected ERα-positive tumors against apoptosis. miR-17-5p was inversely associated with inflammatory cytokines, resulting in the suppression of cancer growth. Recently, it has been reported that miR-144 and miR-126 secreted by adipocytes induced brown differentiation and cancer development [105].

The matrix metalloproteinases (MMPs) family are known to play a crucial role in the invasion and metastasis of cancer cells. MMP-9, MMP-11, and MMP13 are highly prevalent in BC; MMP-9 presents a direct relationship to brain metastases in patients affected by BC [106,107,108] while MMP-11 and MMP13 are promising markers that may be a novel target for the treatment of these patients [109].

Additionally, obesity is involved in BC development through the release of some adipokines, identified as “released hormones” represented by adiponectin, leptin, estrogen, and insulin [97].

Interestingly, adiponectin is the only adipokine to display anti-cancer properties. Moreover, adipocytes are also correlated to chemotherapeutic resistance, resulting in the poorer outcome of treatment. Analysis of the levels of the soluble factors secreted by adipocytes can therefore be useful for the prognosis and evaluation of the effectiveness of cancer therapy [97]. 

Hyperinsulinemia is related to lower IGF-1 expression and aromatase activity, resulting in poor prognosis. Additionally, obesity promotes an inflammatory microenvironment through adipocyte-released cytokines, which facilitates cancer cell progression. Leptin, an adipokine, is strongly concentrated in people with obesity. Leptin promotes BC growth [110,111], acting directly on its receptors, leading to inflammation in the adipocyte microenvironment and increasing the risk of metastatic potential [112]. The activation of the leptin receptor stimulates downstream ERK1/2, AP1, STAT3, PI3K, and MAPK pathways [97]. In addition, leptin also boosts cancer growth/migration via VEGF signaling and HIF-1α stabilization, which induces hypoxia condition in tumors [97].

As briefly introduced before, white FT may produce estrogen through aromatase, which is the most important enzyme for estrogen synthesis in obese post-menopausal females. High levels of estrogen into the breast boost cancer development and metastasis, acting on the ER-membrane pathway including GPCR-like protein, G proteins, MAPK/ERK, and the PI3K/AKT pathway [113]. These pathways contribute to the proliferation and survival of BC via increasing Bcl-2, cyclin D1, and the number of G0/G1 cells [97,113]. 

Additionally, adipocytes may also release several cytokines to regulate the surrounding cells and itself. In vitro co-culture of adipocytes and BCCs resulted in the release of cytokines into the culture supernatant. The outcomes displayed that five (IL6, IL8, IFNγ-inducible protein 10, CCL2, and CCL5) out of 200 cytokines, were significantly increased after one-week of culture, showing immature adipocytes with higher cytokine release potential than mature adipocytes. Cytokines, produced by immature adipocytes, have been shown to ease the cancer starting process and metastasis in BC [114]. Research focused on the follow-up of 534 patients demonstrated that IL6, IL8, and IL10 can be considered as poor prognosis indicators in BC [115]. Many further studies have also emphasized IL6, IL8, CCL2, and CCL5 as promoters of the survival, proliferation, and invasion of BCCs [116,117].

Interestingly, cancer cells may regulate lipidation and lipolysis in adipocytes aiming to provide power and nutrients for cancer growth. Therefore, body weight control in menopausal females should be considered necessary to reduce the BC risk. Given this, a fuller understanding about the functions of adipocytes as well as obesity in BC might reveal novel targets that support the development of new anti-tumor therapy. 

## 8. Oncological Safety of AD-MSCs-Based Therapies

As mentioned previously, many in vitro and in vivo results linked to AD-MSCs demonstrate a shift in the cancer to a more aggressive and invasive phenotype, with regard to an augmented rate of tumor cell proliferation [70]. “While the laboratory data point to AD-MSCs in a pro-carcinogenic role, human clinical studies have yet to confirm the findings of said laboratory studies [62]. Considerable availability, less mobility on the donor site, and fast and uncomplicated harvesting are the most beneficial characteristics exhibited by AD-MSCs versus other types of MSCs; appearing in this way as a useful tool in several neoplastic and non-neoplastic clinical settings [118]. Their capacity to differentiate in several cell types, and additionally stimulate angiogenesis, make them particularly suitable to be used in radiotherapy outcomes and in soft tissue damaged by oncological procedures [119].

The applications of AD-MSC-based therapy have been studied and reported in many oncologic settings, ranging from radiotherapy induced xerostomia [120] to osteosarcoma damage [121,122]; soft tissue reconstruction after sarcoma resection, and laser assisted pulmonary metastasectomy [123]. Most of these include initial singular center experiences that only come with small numbers, retrospective data, short follow-up times, and a generally questionable quality of evidence. In contrast, in breast reconstruction, fat graft was the most frequently performed surgical procedure in the last 10 years, with utility in multiple clinical situations, for example, outcomes of quadrantectomy or mastectomy [124]. 

In an interesting article by Paino et al. [125], the authors co-cultured either SAOS2 osteosarcoma or MCF7 BCCs with human adipose stem cells (hASCs), with the objective of evaluating in vitro and in vivo effects of cancer cells on hASC differentiation. In this report, they observed that both SAOS2 and MCF7 cell lines promoted an increase in hASCs proliferation when compared to hASC alone, but, surprisingly, no modifications in CD90, CD29, CD324, and vimentin expression. Additionally of note, MCF7 and SAOS2 cells induced in hASCs an increase of CD34 expression and stemness genes represented by Sox2, Nanog, Leptin, and OCT3/4 as well as a reduction of pro-angiogenic factors, represented by VEGF, PDGFRα, PDGFRβ, PDGFα, and CD31. SMAD and pSMAD2/3 increased only in hASCs alone. These outcomes suggest that MSCs, under cancer cell induction, do not differentiate in vitro or facilitate the angiogenesis of the cancer in vivo, opening new scenarios in the relationship between cancer and stem cells. 

As known, cancer stem cells (CSCs) play a crucial role in the cancer starting and development process [126]. A given population of CSCs is represented by MSCs that differentiate in meso-dermal cells. Several studies have displayed the MSCs role in the promotion of both pro-tumorigenic or anti-tumorigenic activity [126]. Really, MSCs are conveyed during wound healing process into the area of lesions with the aim to repair the damaged tissues. This process appears to also be correlated with tumorigenesis; for this reason, resident or migrating MSCs may stimulate angiogenesis and cancer development. There is a controversial relationship between MSCs and CSCs since contrasting reports about their respective influences have been reported. 

In a review of Papaccio et al. [126], the authors discussed recent outcomes related to these conflicting results on the influence of normal resident cells and CSCs in cancer development. 

“The major part of information related to the oncological safety of AD-MSCs, AD-SVFs, and fat graft application stems from a sub-group of female patients affected by BC. Fat grafting was expanded upon by Coleman in 1997 [63] and has been put into place as a means to achieve aesthetic and reconstructive goals, which includes oncological breast surgery. The American Society of Plastic Surgeons (ASPS), created in 2007, declared in 2009 that there was no risk of malignancy linked with fat grafting [127]. Despite this, during 2011, a meeting between the ASPS and American Society for Aesthetic Plastic Surgery highlighted a possible recurring risk of malignancy associated with autologous lipofilling and AD-MSCs were promoted [128]. In 2015, the ASPS put out a grade B recommendation stating that fat grafting was not behind an augmented local recurrence risk, despite the fact that a strict adherence to radiological follow-up protocols and an adequate disease-free interval was mandatory (https://www.plasticsurgery.org/Documents/Health-Policy/Principles/principle-2015-post-mastectomy-fat-grafting.pdf). Many authors have studied the effect of fat graft on local cancer for this reason; exploring the distant recurrence within many different clinical works.”

## 9. Controversial Aspects 

AD-SVFs bear a wide cellular constituency, which includes AD-MSCs. Though certainly far from completely understood, the AD-MSCs remain the only known cell that may differentiate along several tissue lineages; as such, it remains a cell of great importance in the development of regenerative cell therapies and tissue-engineering [3,129,130,131,132].

In regenerative plastic surgery, the scientist-surgeon using a system to prepare tissue and isolate cells is viewed by the FDA as an unregulated manufacturer of a biologic. Even when preparing such treatments within the same point-of-care setting, the perspective of the FDA does not change because the same-surgical exemption no longer applies when surgeons or practitioners are “more than minimally manipulating” biomaterials. Therefore, the process of isolating and preparing cells or tissues for regenerative plastic surgery must rely on systems that have been cleared or approved by the FDA for a specific use, not only surgical skill and judgment (i.e., devices cannot be used “off-label”). 

Though several techniques to isolate AD-SVFs from adipose tissue have been developed over the last two decades, to date no standard method exists. Manual manipulation is one such technique, but even when following a precise protocol, outcomes are largely dependent on personal skill and the equipment being used, which makes it difficult to isolate cells of specific CQAs with consistency. 

Without a standardized method and means for isolating an AD-SVF subpopulation with a specific CQA, reproducibly isolating AD-SVFs for targeted regenerative therapies would be difficult, if not simply unreliable. A modular system that can be configured in the form of a variety of point-of-care single-use kits, each of which contains the exact components necessary to conduct a specific protocol, may be best able to provide the standardization that is needed when isolating AD-SVFs and a targeted population of AD-MSCs. Furthermore, with such a system also being modular, it would be possible for it to rapidly accommodate newly discovered CQAs of the AD-MSC target population. Additionally, components of such kits, organized to correlate with each particular protocol, with imposed PQSs and PQLSs may improve the reproducibility of AD-SVF and AD-MSC isolation as well as make possible the standardization of processing biological materials [133]. 

This study establishes the kit’s functionality and efficacy as designed with PQSs and PQLSs as indicated by the resulting data presented herein. 

As end-users (physicians, clinicians, surgeons, and researchers) possess differing skill-set levels, the simple and straightforward use of the kit with inherent PQSs and PQLSs may help reduce variability in the isolation of AD-SVFs and their AD-MSC constituency. 

## 10. Alternative Methods to the Use of AD-SVFs and AD-MSCs

### 10.1. Fat Graft Enrichment with AD-SVFs and Growth Factor

As described, it is possible to isolate an AD-SVF suspension in liquid form through two different type of procedures: enzymatic digestion and mechanical centrifugation. The suspension obtained may be used alone (only AD-SVFs) or in combination with fat graft (AD-SVFs + Fat graft).

In the latter case, the injection of fat graft enriched with AD-SVFs was reported in a different field of regenerative plastic surgery to wound healing [134], breast reconstruction [135,136,137,138,139,140,141,142,143,144], and scars [145,146,147,148]. The biomolecular basis of adipogenic differentiation of AD-MSCs has been reported favoring chondrogenic and osteogenic differentiation [149,150,151,152,153]. The application of AD-SVF suspension alone or in combination with fat graft was reported for the cure of post-traumatic lower extremity ulcers [154].

Alternatively, with the aim of tissue regeneration, it is possible to use, with the same constructive effects, autologous growth factors contained in PRP or synthetic—not-autologous—biomaterial known as dermal substitute (DS). In the first case, growth factors are obtained by the centrifugation of autologous blood, collected in the amount of 9–55 mL, with specific instruments as per the transfusional service protocol. The application of autologous PRP has been reported in the loss of substance with bone exposure [155], in maxillofacial surgery [156], and in plastic surgery [157], particularly for hair loss treatment [158,159,160,161], whereas the use of not-autologous DS has been reported in complex abdominal wall repair [162]. 

With regard to the treatment of hair loss, the use of mesenchymal stem cells obtained with minimal manipulation procedures through centrifugation and the mechanical filtration of scalp tissue has been reported recently in the form of autologous cell therapy based on micro-grafts containing human follicle stem cells (HFSCs) with mesenchymal (HF-MSCs) and epithelial (HF-ESCs) cellular sub-population [163,164,165,166].

### 10.2. Evolution Since “Substitutive Surgery” to “Regenerative Surgery”

The use of autologous AD-MSCs and AD-SVFs aim to regenerate damaged tissues through their use in isolated suspensions or in combination with the adipose tissue from which they have been derived (enrichment procedures). This stems from the necessity to move from “substitutive surgery”, born with transplants, and represented in plastic surgery by the use of osseointegrated implant techniques [167,168,169,170,171] to “regenerative surgery” with the regeneration of organs and tissues induced through autologous cells or where this is not yet possible, the use of autologous tissue grafts [172,173,174].

Recently, several studies have appeared with increasing frequency with the aim to promote regenerative surgery in different fields such as reported, for example, with the use of human periodontal-ligament stem cells in bone regeneration [175,176,177], widening the horizons of regeneration to hitherto unexplored fields.

In each case, as previously reported, without a standardized method for isolating mesenchymal cells with a specific CQA, regenerative therapies would be difficult if not simply unreliable. 

Single-use kits, containing the components necessary to conduct a specific protocol based on minimal manipulation, may be best able to provide the standardization that is needed when isolating AD-SVFs and a targeted population of AD-MSCs. The components of such kits, with imposed PQSs and PQLSs, may improve the reproducibility of AD-SVF and AD-MSC isolation as well as make possible the standardization of processing biological materials.

We are witnessing the definitive sunset of substitutive surgery and, at the same time, we find ourselves at the dawn of regenerative plastic surgery. In both cases, the ambient light is beautiful.

## 11. Cells and Tissue-Sources

Adult MSCs may be harvested from many tissues including from FT in different parts of the body (abdomen, hump, lumbar region, thighs, arms), scalp, bone marrow (BM), and peripheral blood. Previously, BM has been widely recognized as a good source for adult MSCs, since the procedure is effortless and quick and due to the availability of several procedure specific devices. One of the primary limits of this intra-operative stem cell (SC) harvesting techniques was the restricted amount of material harvested and the low amount of cells obtained. This resulted in a SC yield frequency per mono-nucleated cell of one in every 10,000 cells to one in every 100,000 cells [178,179,180,181].

To exceed these issues and to avoid the invasive and painful BM collecting, alternative sources from which to isolate more easily, and in a less invasive way, autologous SCs have been considered.

Between these sources, FT seems to be the most interesting, being able to be collected using a comparatively less invasive technique like liposuction, obtaining larger cellular yields compared to BM, providing a cell population characterized by multi-lineage separation potential [182]. In fact, FT harvest is much easier and less painful for the patient when compared with BM harvesting, where it is necessary to use a trocar to drill through the iliac crest [182]. A few works have displayed differences between SCs obtained from various sources [183,184,185]. FT must be considered as a true alternative to BM for intra-operative autologous stem cell-based therapy use on the basis of the expansion potential of the SCs. A limitation of the FT use could be represented by limited or minimal FT amount in thin patients. Really, the high frequency of AD-MSCs (their occurrence is 100 to 300 times higher than in BM, and the number of SCs that can be counted per unit volume of fat collected is approximately 10-fold greater than that from BM) could allow the collection of small FT deposits and, at the same time, be adequate for SC isolation. The donor site area and the surgical technique used to collect FT may affect the cellular yield. For example, FT collected from the abdomen through resection or liposuction yields more SCs in contrast with ultrasound-assisted liposuction and with the FT collected from the hip/thigh district [186].

For the scalp [163,164,165,166], as previously mentioned, Gentile et al. developed different procedures to separate human adult adipose-derived follicle SCs (HD-AFSCs), according to minimal manipulation rules, based on the centrifugation of scalp tissue fragments containing FT, dermal, and human hair follicles without cellular expansion or culture. Specifically, the authors reported, for the first time, the outcomes obtained in a hair re-growth study using a “Gentile procedure” [167] that was used to obtain autologous micro-grafts containing human hair follicle MSCs (HF-MSCs) from centrifugation of scalp biopsies, easily accessible for use in patients affected by AGA. The micro-graft units were obtained by the disaggregation of several 2 mm punch biopsies and the selection of a cell populace with a diameter of 80–140 microns.

The clinical use of HD-AFSCs and HF-MSCs to enhance hair re-growth has not been satisfactorily considered. 

In their study, the authors displayed the amount of CD44+ cells—HF-MSCs—from the dermal papilla, and the level of CD200+ cells—hair follicle epithelial-SCs—(HF-ESCs) from the bulge, obtained by means of the disaggregation of several punch tests [163,164,165,166]. 

In each case, the advantages of AD-MSCs over BM-SCs such as being available and less discomfort during harvest have made them a good alternative instead of BM-SCs in tissue engineering. AD-MSCs from buccal fat pad (BFP), as an easily harvestable and accessible source, have gained interest for use in bone regeneration in the maxillofacial region. In a study by Rezai Rad et al. [167], AD-MSCs have been isolated from different sites BFP (BFP-AD-MSCs), abdomen (abdomen adipose-derived mesenchymal stem cells (Ab-AD-MSCs), hip (hip adipose-derived mesenchymal stem cells (Hip-AD-MSCs) from one individual, and compared the morphology, surface marker expression, osteogenic differentiation capability, and growth rate. 

Among them, BFP-AD-MSCs displayed the highest proliferation rate with the shortest doubling time and also expressed vascular endothelial markers including CD146 and CD34.

Additionally, among them, the expression of osteogenic markers were higher in BFP-AD-MSCs, suggesting that BFP-AD-MSCs may represent an interesting source of MSCs for bone defect treatment tissue engineering [187].

Another study by Ardeshirylajimi A et al. [188] reported on the potential differences in AD-MSC proliferation properties by comparing aspirates from the abdomen and hump. They reported that AD-MSCs from both the abdominal region and the hump exhibited spindle-shaped and fibroblast-like morphology with hump-derived AD-MSCs, being smaller in size and narrower in overall appearance than abdominal AD-MSCs. Abdominal AD-MSCs required a greater time for proliferation than the hump-derived cells. These results were further confirmed with a tetrazolium-based colorimetric assay (MTT), which displayed a greater cell proliferation rate for hump AD-MSCs than for the abdominal region. This study revealed that AD-MSCs may be obtained from different anatomical regions, although ASCs from the hump fat region may be the ideal stem cell sources for use in cell-based therapies. The fat harvesting site is an important determinant of proliferation and pluripotency of AD-MSCs.

## 12. Conclusions

Using a kit with the PQSs and PQLSs described in this study enabled the isolation of AD-MSCs that meet the consensus identity criteria. As the discovery of new CQAs of ASCs evolves with respect to purity and potency, adjustments to these benchmark PQSs and PQLs will hopefully isolate AD-MSCs of various CQAs with greater reproducibility, quality, and safety. Confirmatory studies will need to be completed.

## Figures and Tables

**Table 1:** Comparative analysis of the results obtained with enzymatic and non-enzymatic procedures.

Procedure	Method	Field	Yield	References
AdiStem™ cell isolation kit in combination with PRP (i.e., platelet-rich plasma)	Enzymatic	Joint regeneration	12 Å~10^6^/mL fat (versus standard isolation procedure 10 Å~10^6^ cells/mL fat)	[24,25]
		Endobronchially infusion in human patients affected by idiopathic pulmonary fibrosis (IPF)		[26,27]
Celution^®^ 800/CRS system	Enzymatic	Reduction of fat resorption, increasing the angiogenesis	2.95 × 10^5^ cells/mL with a viability of 86.6%	[29,31]
		Improving hand disability, reducing pain in systemic sclerosis		[32]
		Transurethrally infiltrated, resulted in reduction of male stress urinary incontinence		[33]
		Improving breast contour		[34]
		Outcomes of breast reconstruction		[42,62]
		--	2.41 × 10^5^ cells/g	[30]
Multi-Station	Enzymatic		1.07 × 10^5^ cells/g	[30]
Lipo-Kit GT	Enzymatic	Outcomes of breast reconstruction	0.35 × 10^5^ cells/g	[30,42]
CHA STATION™	Enzymatic		0.05 × 10^5^ cells/g	[30]
GID SVF-1™	Enzymatic		7.19 ± 2.11 × 10^5^ cells/mL	[35]
Sceldis^®^ device in combination with PRP, hyaluronic acid, and CaCl^2+^	Enzymatic	Treatment of knee pain		[36,37]
Method of Khan et al.	Enzymatic		~6 × 10^5^ cells/mL (66 % viability) versus ~1 × 10^6^ cells/mL (51% viability)	[38]
HuriCell device	Enzymatic	Pre-clinical model of focal cerebral ischemia treatment, displaying neuro-protective effects		[39]
Stubbers and Coleman procedure	Enzymatic		4.9 × 10^6^–24.7 × 10^6^ cells/100 g	[40]
Tissue Genesis Icellator Cell Isolation System	Enzymatic	Face and breast augmentation or reconstruction		[41]
Puregraft^®^	Filtration, washing and purification			[43,44]
Revolve™	Filtration, washing and purification			---
GID 700™	Washing and purification	Significant reduction amounts of lactate dehydrogenase, triglycerides and hematocrit maintaining the adipose graft osmolarity		[45]
LipiVage™	Purification, Filtration	Endothelial and mesenchymal progenitor cells maintaining their differentiation capacity when used as fibrin spray		[46]
		High number of adipocytes and a high level of intracellular enzyme (glycerol-3-phophatase dehydrogenase (G3PDH)		[47]
Lipogems^®^	Micro-fragmentation	Higher concentration of mature pericytes, AD-MSCs, exosomes and lower quantity of hematopoietic cells		[48,49,52]
		Paracrine and arteriogenic functions for the rescue of ischemic limb		[50]
		Improved efficient direct multi-lineage reprogramming in human skin fibroblasts		[51]
		Orthognatic surgery		[53]
		Osteointegration		[54]
		Fecal incontinence’s symptoms		[55]
System for endothelial cells of Hu et al.	homogenizing fat tissue		1.12–2.13 Å~106 cells	[56]
System for AD-SVFs isolation of Victor	Ultrasonic cavitation		1.67–2.24 Å~107 cells with a viability of 97.1–98.9%	[57]
System for dissociation of fat tissue of Bright et al.	Dissociation of fat tissue by lysing mature adipocytes applying ultrasonic cavitation	Osteoarthritis (knee, hip)	2–4 million cells/1 g fat	[58,59]
		Chronic migraine		[59]
Schafer System to isolate fat derived cells	Ultrasonic energy/acoustic standing wave	(<50 μm) but more vital pre-adipocytes, stimulating the angiogenesis		[60]
Gimble et al. procedure	Shaking, washing		2.5 Å~106 cells per 100 mL fat	[61]

## References

[1] Coelho M.B., Cabral J.M., Karp J.M. (2012). Intraoperative stem cell therapy. Annu. Rev. Biomed. Eng..

[2] Bourin P., Bunnell B.A., Casteilla L., Dominici M., Katz A.J., March K.L., Redl H., Rubin J.P., Yoshimura K., Gimble J.M. (2013). Stromal cells from the adipose tissue-derived stromal vascular fraction and culture expanded adipose tissue-derived stromal/stem cells: A joint statement of the International Federation for Adipose Therapeutics and Science (IFATS) and the International Society for Cellular Therapy (ISCT). Cytotherapy.

[3] Zuk P.A., Zhu M., Ashjian P., de Ugarte D.A., Huang J.I., Mizuno H., Alfonso Z.C., Fraser J.K., Benhaim P., Hedrick M.H. (2002). Human adipose tissue is a source of multipotent stem cells. Mol. Biol. Cell.

[4] Gimble J., Guilak F. (2003). Adipose-derived adult stem cells: Isolation, characterization, and differentiation potential. Cytotherapy.

[5] Carvalho P.P., Gimble J.M., Dias I.R., Gomes M.E., Reis R.L. (2013). Xenofree enzymatic products for the isolation of human adipose-derived stromal/stem cells. Tissue Eng. Part C Methods.

[6] Patrikoski M., Juntunen M., Boucher S., Campbell A., Vemuri M.C., Mannerstrom B., Miettinen S. (2013). Development of fully defined xeno-free culture system for the preparation and propagation of cell therapy-compliant human adipose stem cells. Stem Cell Res. Ther..

[7] Aguena M., Fanganiello R.D., Tissiani L.A., Ishiy F.A., Atique R., Alonso N., Passos-Bueno M.R. (2012). Optimization of parameters for a more efficient use of adipose-derived stem cells in regenerative medicine therapies. Stem Cells Int..

[8] Yang X.F., He X., He J., Zhang L.H., Su X.J., Dong Z.Y., Xu Y.J., Li Y., Li Y.L. (2011). High efficient isolation and systematic identification of human adipose-derived mesenchymal stem cells. J. Biomed. Sci..

[9] Markarian C.F., Frey G.Z., Silveira M.D., Chem E.M., Milani A.R., Ely P.B., Horn A.P., Nardi N.B., Camassola M. (2014). Isolation of adipose-derived stem cells: A comparison among different methods. Biotechnol. Lett..

[10] Philips B.J., Marra K.G., Rubin J.P. (2012). Adipose stem cell-based soft tissue regeneration. Expert Opin. Biol. Ther..

[11] Thirumala S., Gimble J.M., Devireddy R.V. (2010). Cryopreservation of stromal vascular fraction of adipose tissue in a serum-free freezing medium. J. Tissue Eng. Regen. Med..

[12] Zimmerlin L., Donnenberg V.S., Pfeifer M.E., Meyer E.M., Peault B., Rubin J.P., Donnenberg A.D. (2010). Stromal vascular progenitors in adult human adipose tissue. Cytom. Part A J. Int. Soc. Anal. Cytol..

[13] Kirkpatrick C.J., Melzner I., Goller T. (1985). Comparative effects of trypsin, collagenase and mechanical harvesting on cell membrane lipids studied in monolayercultured endothelial cells and a green monkey kidney cell line. Biochim. Biophys. Acta.

[14] Stadler G., Hennerbichler S., Lindenmair A., Peterbauer A., Hofer K., van Griensven M., Gabriel C., Redl H., Wolbank S. (2008). Phenotypic shift of human amniotic epithelial cells in culture is associated with reduced osteogenic differentiation in vitro. Cytotherapy.

[15] Kakagia D., Pallua N. (2014). Autologous fat grafting: In search of the optimal technique. Surg. Innov..

[16] Garza R.M., Paik K.J., Chung M.T., Duscher D., Gurtner G.C., Longaker M.T., Wan D.C. (2014). Studies in fat grafting: Part III. Fat grafting irradiated tissue—Improved skin quality and decreased fat graft retention. Plast. Reconstr. Surg..

[17] Stillaert F.B., di Bartolo C., Hunt J.A., Rhodes N.P., Tognana E., Monstrey S., Blondeel P.N. (2008). Human clinical experience with adipose precursor cells seeded on hyaluronic acid-based spongy scaffolds. Biomaterials.

[18] Matsumoto D., Sato K., Gonda K., Takaki Y., Shigeura T., Sato T., Aiba-Kojima E., Iizuka F., Inoue K., Suga H. (2006). Cellassisted lipotransfer: Supportive use of human adipose-derived cells for soft tissue augmentation with lipoinjection. Tissue Eng..

[19] Holnthoner W., Hohenegger K., Husa A.M., Muehleder S., Meinl A., Peterbauer-Scherb A., Redl H. (2015). Adipose-derived stem cells induce vascular tube formation of outgrowth endothelial cells in a fibrin matrix. J. Tissue Eng. Regen. Med..

[20] Gentile P., Orlandi A., Scioli M.G., di Pasquali C., Bocchini I., Curcio C.B., Floris M., Fiaschetti V., Floris R., Cervell V. (2012). A comparative translational study: The combined use of enhanced stromal vascular fraction and platelet-rich plasma improves fat grafting maintenance in breast reconstruction. Stem Cells Transl. Med..

[21] Jiang A., Li M., Duan W., Dong Y., Wang Y. (2015). Improvement of the survival of human autologous fat transplantation by adipose-derived stem-cellsassisted lipotransfer combined with bFGF. Sci. World J..

[22] Luo S., Hao L., Li X., Yu D., Diao Z., Ren L., Xu H. (2013). Adipose tissue-derived stem cells treated with estradiol enhance survival of autologous fat transplants. Tohoku J. Exp. Med..

[23] Li L., Pan S., Ni B., Lin Y. (2014). Improvement in autologous human fat transplant survival with SVF plus VEGF-PLA nano-sustained release microspheres. Cell Biol. Int..

[24] van Pham P., Hong-Thien Bui K., Quoc Ngo D., Tan Khuat L., Kim P.N. (2013). Transplantation of nonexpanded adipose stromal vascular fraction and platelet-rich plasma for articular cartilage injury treatment in mice model. J. Med. Eng..

[25] Paspaliaris B., Thornton J.A.F. (2014). Methods and Apparatuses for Isolating and Preparing Stem Cells. U.S. Patent.

[26] Tzouvelekis A., Koliakos G., Ntolios P., Baira I., Bouros E., Oikonomou A., Zissimopoulos A., Kolios G., Kakagia D., Paspaliaris V. (2011). Stem cell therapy for idiopathic pulmonary fibrosis: A protocol proposal. J. Transl. Med..

[27] Tzouvelekis A., Paspaliaris V., Koliakos G., Ntolios P., Bouros E., Oikonomou A., Zissimopoulos A., Boussios N., Dardzinski B., Gritzalis D. (2013). A prospective, non-randomized, no placebo-controlled, phase Ib clinical trial to study the safety of the adipose derived stromal cells-stromal vascular fraction in idiopathic pulmonary fibrosis. J. Transl. Med..

[28] Michalek J., Moster R., Lukac L., Proefrock K., Petrasovic M., Rybar J., Capkova M., Chaloupka A., Darinskas A., Michalek J. (2015). Autologous adipose tissue-derived stromal vascular fraction cells application in patients with osteoarthritis. Cell Transpl..

[29] Lin K., Matsubara Y., Masuda Y., Togashi K., Ohno T., Tamura T., Toyoshima Y., Sugimachi K., Toyoda M., Marc H. (2008). Characterization of adipose tissue-derived cells isolated with the Celution system. Cytotherapy.

[30] Aronowitz J.A., Ellenhorn J.D. (2013). Adipose stromal vascular fraction isolation: A head-to-head comparison of four commercial cell separation systems. Plast. Reconstr. Surg..

[31] Kakudo N., Tanaka Y., Morimoto N., Ogawa T., Kushida S., Hara T., Kusumoto K. (2013). Adipose-derived regenerative cell (ADRC)-enriched fat grafting: Optimal cell concentration and effects on grafted fat characteristics. J. Transl. Med..

[32] Granel B., Daumas A., Jouve E., Harle J.R., Nguyen P.S., Chabannon C., Colavolpe N., Reynier J.C., Truillet R., Mallet S. (2015). Safety, tolerability and potential efficacy of injection of autologous adipose-derived stromal vascular fraction in the fingers of patients with systemic sclerosis: An open-label phase I trial. Ann. Rheum. Dis..

[33] Gotoh M., Yamamoto T., Kato M., Majima T., Toriyama K., Kamei Y., Matsukawa Y., Hirakawa A., Funahashi Y. (2014). Regenerative treatment of male stress urinary incontinence by periurethral injection of autologous adipose-derived regenerative cells: 1-year outcomes in 11 patients. Int. J. Urol..

[34] Perez-Cano R., Vranckx J.J., Lasso J.M., Calabrese C., Merck B., Milstein A.M., Sassoon E., Delay E., Weiler-Mithoff E.M. (2012). Prospective trial of adipose-derived regenerative cell (ADRC)-enriched fat grafting for partial mastectomy defects: The RESTORE-2 trial. Eur. J. Surg. Oncol..

[35] Vilaboa S.D.A., Navarro-Palou M., Llull R. (2014). Age influence on stromal vascular fraction cell yield obtained from human lipoaspirates. Cytotherapy.

[36] Pak J., Lee J.H., Lee S.H. (2014). Regenerative repair of damaged meniscus with autologous adipose tissue-derived stem cells. BioMed Res. Int..

[37] Pak J., Chang J.J., Lee J.H., Lee S.H. (2013). Safety reporting on implantation of autologous adipose tissue-derived stem cells with platelet-rich plasma into human articular joints. BMC Musculoskelet. Disord..

[38] Khan Z.A., Dulgar-Tulloch A.J., Rakuff S., Shoemaker P.A., Kvam E.L., Chen X., Roy J. (2012). Automated Systems and Methods for Isolating Regenerative Cells from Adipose Tissue. U.S. Patent.

[39] Kaengkan P., Baek S.E., Kim J.Y., Kam K.Y., Do B.R., Lee E.S., Kang S.G. (2013). Administration of mesenchymal stem cells and ziprasidone enhanced amelioration of ischemic brain damage in rats. Mol. Cells.

[40] Stubbers R., Coleman M.E. (2015). Apparatus and Methods for Cell Isolation. U.S. Patent.

[41] Doi K., Tanaka S., Iida H., Eto H., Kato H., Aoi N., Kuno S., Hirohi T., Yoshimura K. (2013). Stromal vascular fraction isolated from lipo-aspirates using an automated processing system: Bench and bed analysis. J. Tissue Eng. Regen. Med..

[42] Domenis R., Lazzaro L., Calabrese S., Mangoni D., Gallelli A., Bourkoula E., Manini I., Bergamin N., Toffoletto B., Beltrami C.A. (2015). Adipose tissue derived stem cells: In vitro and in vivo analysis of a standard and three commercially available cell-assisted lipotransfer techniques. Stem Cell Res. Ther..

[43] Schafer M.E., Hicok K.C., Mills D.C., Cohen S.R., Chao J.J. (2013). Acute adipocyte viability after third-generation ultrasound-assisted liposuction. Aesthet. Surg. J..

[44] Zhu M., Cohen S.R., Hicok K.C., Shanahan R.K., Strem B.M., Yu J.C., Arm D.M., Fraser J.K. (2013). Comparison of three different fat graft preparation methods: Gravity separation, centrifugation, and simultaneous washing with filtration in a closed system. Plast. Reconstr. Surg..

[45] Dos-Anjos Vilaboa S., Llull R., Mendel T.A. (2013). Returning fat grafts to physiologic conditions using washing. Plast. Reconstr. Surg..

[46] Zimmerlin L., Rubin J.P., Pfeifer M.E., Moore L.R., Donnenberg V.S., Donnenberg A.D. (2013). Human adipose stromal vascular cell delivery in a fibrin spray. Cytotherapy.

[47] Ferguson R.E., Cui X., Fink B.F., Vasconez H.C., Pu L.L. (2008). The viability of autologous fat grafts harvested with the LipiVage system: A comparative study. Ann. Plast. Surg..

[48] Bianchi F., Maioli M., Leonardi E., Olivi E., Pasquinelli G., Valente S., Mendez A.J., Ricordi C., Raffaini M., Tremolada C. (2013). A new nonenzymatic method and device to obtain a fat tissue derivative highly enriched in pericyte-like elements by mild mechanical forces from human lipoaspirates. Cell Transplant..

[49] García-Contreras M., Messaggio F., Jimenez O., Mendez A. (2015). Differences in exosome content of human adipose tissue processed by non-enzymatic and enzymatic methods. CellR4.

[50] Bianchi F., Olivi E., Baldassarre M., Giannone F.A., Laggetta M., Valente S., Cavallini C., Tassinari R., Canaider S., Pasquinelli G. (2014). Lipogems, a new modality of fat tissue handling to enhance tissue repair in chronic hind limb ischemia. CellR4.

[51] Maioli M., Rinaldi S., Santaniello S., Castagna A., Pigliaru G., Delitala A., Bianchi F., Tremolada C., Fontani V., Ventura C. (2014). Radioelectric asymmetric conveyed fields and human adipose-derived stem cells obtained with a non enzymatic method and device: A novel approach to multipotency. Cell Transplant..

[52] Carelli S., Messaggio F., Canazza A., Hebda D.M., Caremoli F., Latorre E., Grimoldi M.G., Colli M., Bulfamante G., Tremolada C. (2015). Characteristics and properties of mesenchymal stem cells derived from micro-fragmented adipose tissue. Cell Transplant..

[53] Raffaini M., Tremolada C. (2014). Micro fractured and purified adipose tissue graft (Lipogems^®^) can improve the orthognathic surgery outcomes both aesthetically and in postoperative healing. CellR4.

[54] Benzi R., Marfia G., Bosetti M., Beltrami G., Magri A.S., Versari S., Tremolada C. (2015). Microfractured lipoaspirate may help oral bone and soft tissue regeneration: A case report. CellR4.

[55] Giori A., Tremolada C., Vailati R., Navone S.E., Marfia G., Caplan A.I. (2015). Recovery of function in anal incontinence after micro-fragmented fat graft (Lipogems^®^) injection: Two years follow up of the first 5 cases. CellR4.

[56] Hu C.B., Myers K.E., Peterson R.C. (2000). Devices for Harvesting and Homogenizing Adipose Tissue Containing Autologous Endothelial Cells. U.S. Patent.

[57] Victor S. (2014). Isolation of Stromal Vascular Fraction from Adipose Tissue Obtained from Postmortem Source Using Ultrasonic Cavitation. WO Patent.

[58] Bright R., Bright P., Hansen B., Thomas W. (2014). Isolation of Stem Cells from Adipose Tissue by Ultrasonic Cavitation, and Methods of Use. WO Patent.

[59] Bright R., Bright M., Bright P., Hayne S., Thomas W.D. (2014). Migraine and tensiontype headache treated with stromal vascular fraction: A case series. J. Med. Case Rep..

[60] Schafer M.E. (2013). Selective Lysing of Cells Using Ultrasound. U.S. Patent.

[61] Gimble J.M., Shah F.S., Wu X. (2014). Non-Enzymatic Method for Isolating Human Adipose-Derived Stromal Stem Cells. U.S. Patent.

[62] O’Halloran N., Courtney D., Kerin M.J., Lowery A.J. (2017). Adipose-derived stem cells in novel approaches to breast reconstruction: Their suitability for tissue engineering and oncological safety. Breast Cancer Basic Clin. Res..

[63] Coleman S.R. (1997). Facial recontouring with lipostructure. Clin. Plast. Surg..

[64] Kling R.E., Mehrara B.J., Pusic A.L., Young V.L., Hume K.M., Crotty C.A., Rubin J.P. (2013). Trends in autologous fat grafting to the breast: A national survey of the american society of plastic surgeons. Plast. Reconstr. Surg..

[65] Toyserkani N.M., Jorgensen M.G., Tabatabaeifar S., Jensen C.H., Sheikh S.P., Sorensen J.A. (2017). Concise review: A safety assessment of adipose-derived cell therapy in clinical trials: A systematic review of reported adverse events. Stem Cells Transl. Med..

[66] Pennati A., Riggio E., Marano G., Biganzoli E. (2018). Autologous fat grafting after sarcoma surgery: Evaluation of oncological safety. J. Plast. Reconstr. Aesthet. Surg..

[67] Jurado M., de La Mata C., Ruiz-Garcia A., Lopez-Fernandez E., Espinosa O., Remigia M.J., Moratalla L., Goterris R., Garcia-Martin P., Ruiz-Cabello F. (2017). Adipose tissue-derived mesenchymal stromal cells as part of therapy for chronic graft-versus-host disease: A phase I/II study. Cytotherapy.

[68] Rigotti G., Marchi A., Galie M., Baroni G., Benati D., Krampera M., Pasini A., Sbarbati A. (2007). Clinical treatment of radiotherapy tissue damage by lipoaspirate transplant: A healing process mediated by adipose-derived adult stem cells. Plast. Reconstr. Surg..

[69] Zhang Y., Bellows C.F., Kolonin M.G. (2010). Adipose tissue-derived progenitor cells and cancer. World J. Stem Cells.

[70] Freese K.E., Kokai L., Edwards R.P., Philips B.J., Sheikh M.A., Kelley J., Comerci J., Marra K.G., Rubin J.P., Linkov F. (2015). Adipose-derived stems cells and their role in human cancer development, growth, progression, and metastasis: A systematic review. Cancer Res..

[71] Li W., Xu H., Qian C. (2017). c-Kit-Positive adipose tissue-derived mesenchymal stem cells promote the growth and angiogenesis of breast cancer. BioMed Res. Int..

[72] Salha S., Gehmert S., Brebant V., Anker A., Loibl M., Prantl L., Gehmert S. (2018). PDGF regulated migration of mesenchymal stem cells towards malignancy acts via the PI3K signaling pathway. Clin. Hemorheol. Microcirc..

[73] Preisner F., Leimer U., Sandmann S., Zoernig I., Germann G., Koellensperger E. (2018). Impact of human adipose tissue-derived stem cells on malignant melanoma cells in an in vitro co-culture model. Stem Cell Rev..

[74] Razmkhah M., Mansourabadi Z., Mohtasebi M.S., Talei A.R., Ghaderi A. (2018). Cancer and normal adipose-derived mesenchymal stem cells (ASCs): Do they have differential effects on tumor and immune cells?. Cell Biol. Int..

[75] Xishan Z., Bin Z., Haiyue Z., Xiaowei D., Jingwen B., Guojun Z. (2015). Jagged-2 enhances immunomodulatory activity in adipose derived mesenchymal stem cells. Sci. Rep..

[76] Zimmerlin L., Park T.S., Zambidis E.T., Donnenberg V.S., Donnenberg A.D. (2013). Mesenchymal stem cell secretome and regenerative therapy after cancer. Biochimie.

[77] Banas A., Teratani T., Yamamoto Y., Tokuhara M., Takeshita F., Osaki M., Kawamata M., Kato T., Okochi H., Ochiya T. (2008). IFATS collection: In vivo therapeutic potential of human adipose tissue mesenchymal stem cells after transplantation into mice with liver injury. Stem Cells.

[78] Anjanappa M., Burnett R., Zieger M.A., Merfeld-Clauss S., Wooden W., March K., Tholpady S., Nakshatri H. (2016). Distinct effects of adipose-derived stem cells and adipocytes on normal and cancer cell hierarchy. Mol. Cancer Res..

[79] Lu Y., Yang Y., Liu Y., Hao Y., Zhang Y., Hu Y., Jiang L., Gong Y., Wu K., Liu Y. (2017). Upregulation of PAG1/Cbp contributes to adipose-derived mesenchymal stem cells promoted tumor progression and chemoresistance in breast cancer. Biochem. Biophys. Res. Commun..

[80] Campbell K. (2018). Contribution of epithelial-mesenchymal transitions to organogenesis and cancer metastasis. Curr. Opin. Cell Biol..

[81] Ritter A., Friemel A., Fornoff F., Adjan M., Solbach C., Yuan J., Louwen F. (2015). Characterization of adipose-derived stem cells from subcutaneous and visceral adipose tissues and their function in breast cancer cells. Oncotarget.

[82] Kucerova L., Skolekova S., Matuskova M., Bohac M., Kozovska Z. (2013). Altered features and increased chemosensitivity of human breast cancer cells mediated by adipose tissue-derived mesenchymal stromal cells. BMC Cancer.

[83] Strong A.L., Ohlstein J.F., Biagas B.A., Rhodes L.V., Pei D.T., Tucker H.A., Llamas C., Bowles A.C., Dutreil M.F., Zhang S. (2015). Leptin produced by obese adipose stromal/stem cells enhances proliferation and metastasis of estrogen receptor positive breast cancers. Breast Cancer Res..

[84] Visweswaran M., Keane K.N., Arfuso F., Dilley R.J., Newsholme P., Dharmarajan A. (2018). The influence of breast tumour-derived factors and Wnt antagonism on the transformation of adipose-derived mesenchymal stem cells into tumour-associated fibroblasts. Cancer Microenviron..

[85] Song Y.H., Warncke C., Choi S.J., Choi S., Chiou A.E., Ling L., Liu H.Y., Daniel S., Antonyak M.A., Cerione R.A. (2017). Breast cancer-derived extracellular vesicles stimulate myofibroblast differentiation and pro-angiogenic behavior of adipose stem cells. Matrix Biol..

[86] Lin R., Wang S., Zhao R.C. (2013). Exosomes from human adipose-derived mesenchymal stem cells promote migration through Wnt signaling pathway in a breast cancer cell model. Mol. Cell. Biochem..

[87] Stamatopoulos A., Stamatopoulos T., Gamie Z., Kenanidis E., Ribeiro R.D.C., Rankin K.S., Gerrand C., Dalgarno K., Tsiridis E. (2019). Mesenchymal stromal cells for bone sarcoma treatment: Roadmap to clinical practice. J. Bone Oncol..

[88] Ghosh S., Hughes D., Parma D.L., Ramirez A., Li R. (2014). Association of obesity and circulating adipose stromal cells among breast cancer survivors. Mol. Biol. Rep..

[89] Bellows C.F., Zhang Y., Chen J., Frazier M.L., Kolonin M.G. (2011). Circulation of progenitor cells in obese and lean colorectal cancer patients. Cancer Epidemiol. Prev. Biomark..

[90] Ribeiro R., Monteiro C., Silvestre R., Castela A., Coutinho H., Fraga A., Principe P., Lobato C., Costa C., Cordeiro-da-Silva A. (2012). Human periprostatic white adipose tissue is rich in stromal progenitor cells and a potential source of prostate tumor stroma. Exp. Biol. Med..

[91] Chulpanova D.S., Kitaeva K.V., Tazetdinova L.G., James V., Rizvanov A.A., Solovyeva V.V. (2018). Application of mesenchymal stem cells for therapeutic agent delivery in anti-tumor treatment. Front. Pharmacol..

[92] Locke M., Windsor J., Dunbar P.R. (2009). Human adipose-derived stem cells: Isolation, characterization and applications in surgery. ANZ J. Surg..

[93] Martin-Padura I., Gregato G., Marighetti P., Mancuso P., Calleri A., Corsini C., Pruneri G., Manzotti M., Lohsiriwat V., Rietjens M. (2012). The white adipose tissue used in lipotransfer procedures is a rich reservoir of CD34^+^ progenitors able to promote cancer progression. Cancer Res..

[94] Hass R., Otte A. (2012). Mesenchymal stem cells as all-round supporters in a normal and neoplastic microenvironment. Cell Commun. Signal..

[95] Gao Z., Zhang L., Hu J., Sun Y. (2013). Mesenchymal stem cells: A potential targeted-delivery vehicle for anti-cancer drug, loaded nanoparticles. Nanomed. Nanotechnol. Biol. Med..

[96] Schweizer R., Tsuji W., Gorantla V.S., Marra K.G., Rubin J.P., Plock J.A. (2015). The role of adipose-derived stem cells in breast cancer progression and metastasis. Stem Cells Int..

[97] Chu D.T., Phuong T.N.T., Tien N.L.B., Tran D.K., Nguyen T.T., Thanh V.V., Quang T.L., Minh L.B., Pham V.H., Ngoc V.T.N. (2019). The effects of adipocytes on the regulation of breast cancer in the tumor microenvironment: An update. Cells.

[98] Soto-Guzman A., Navarro-Tito N., Castro-Sanchez L., Martinez-Orozco R., Salazar E.P. (2010). Oleic acid promotes MMP-9 secretion and invasion in breast cancer cells. Clin. Exp. Metastasis.

[99] Hardy S., Langelier Y., Prentki M. (2000). Oleate activates phosphatidylinositol 3-kinase and promotes proliferation and reduces apoptosis of MDA-MB-231 breast cancer cells, whereas palmitate has opposite effects 1. Cancer Res..

[100] Cleofas M.-M., Alejandra O.-M., Christian G.-R., Pedro C.-R., Eduardo Perez S. (2019). Oleic acid induces migration through a FFAR1/4, EGFR and AKT-dependent pathway in breast cancer cells. Endocr. Connect..

[101] Touvier M., Fassier P., His M., Norat T., Chan D.S.M., Blacher J., Hercberg S., Galan P., Druesne-Pecollo N., Latino-Martel P. (2015). Cholesterol and breast cancer risk: A systematic review and meta-analysis of prospective studies. Br. J. Nutr..

[102] His M., Dartois L., Fagherazzi G., Boutten A., Dupré T., Mesrine S., Boutron-Ruault M.-C., Clavel-Chapelon F., Dossus L. (2017). Associations between serum lipids and breast cancer incidence and survival in the E3N prospective cohort study. Cancer Causes Control.

[103] Thomou T., Mori M.A., Dreyfuss J.M., Konishi M., Sakaguchi M., Wolfrum C., Rao T.N., Winnay J.N., Garcia-Martin R., Grinspoon S.K. (2017). Adipose-derived circulating miRNAs regulate gene expression in other tissues. Nature.

[104] Rajarajan D., Selvarajan S., Raja M.R.C., Mahapatra S.K., Kasiappan R. (2019). Genome-wide analysis reveals miR-3184-5p and miR-181c-3p as a critical regulator for adipocytes-associated breast cancer. J. Cell. Physiol..

[105] Wu Q., Li J., Li Z., Sun S., Zhu S., Wang L., Wu J., Yuan J., Zhang Y., Sun S. (2019). Exosomes from the tumour–adipocyte interplay stimulate beige/brown differentiation and reprogram metabolism in stromal adipocytes to promote tumour progression. J. Exp. Clin. Cancer Res..

[106] Huang H. (2018). Matrix metalloproteinase-9 (MMP-9) as a cancer biomarker and MMP-9 biosensors: Recent advances. Sensors.

[107] Gautam J., Banskota S., Lee H., Lee Y.J., Jeon Y.H., Kim J.A., Jeong B.S. (2018). Down-regulation of cathepsin S and matrix metalloproteinase-9 via Src, a non-receptor tyrosine kinase, suppresses triple-negative breast cancer growth and metastasis. Exp. Mol. Med..

[108] Lafleur M.A., Drew A.F., de Sousa E.L., Blick T., Bills M., Walker E.C., Williams E.D., Waltham M., Thompson E.W. (2005). Upregulation of matrix metalloproteinases (MMPs) in breast cancer xenografts: A major induction of stromal MMP-13. Int. J. Cancer.

[109] Roscilli G., Cappelletti M., de Vitis C., Ciliberto G., di Napoli A., Ruco L., Mancini R., Aurisicchio L. (2014). Circulating MMP11 and specific antibody immune response in breast and prostate cancer patients. J. Transl. Med..

[110] Vaisse C., Halaas J.L., Horvath C.M., Darnell J.E., Stoffel M., Friedman J.M. (1996). Leptin activation of Stat3 in the hypothalamus of wild–type and ob/ob mice but not db/db mice. Nat. Genet..

[111] Guo S., Liu M., Wang G., Torroella-Kouri M., Gonzalez-Perez R.R. (2012). Oncogenic role and therapeutic target of leptin signaling in breast cancer and cancer stem cells. Biochim. Biophys. Acta.

[112] Delort L., Rossary A., Farges M.C., Vasson M.P., Caldefie-Chézet F. (2015). Leptin, adipocytes and breast cancer: Focus on inflammation and anti-tumor immunity. Life Sci..

[113] Wong C.W., McNally C., Nickbarg E., Komm B.S., Cheskis B.J. (2002). Estrogen receptor-interacting protein that modulates its nongenomic activity-crosstalk with Src/Erk phosphorylation cascade. Proc. Natl. Acad. Sci. USA.

[114] Picon-Ruiz M., Pan C., Drews-Elger K., Jang K., Besser A.H., Zhao D., Morata-Tarifa C., Kim M., Ince T.A., Azzam D.J. (2016). Interactions between adipocytes and breast cancer cells stimulate cytokine production and drive Src/Sox2/miR-302b–mediated malignant progression. Cancer Res..

[115] Wang H., Yang X. (2017). Association between serum cytokines and progression of breast cancer in Chinese population. Medicine.

[116] Waugh D.J.J., Wilson C. (2008). The interleukin-8 pathway in cancer. Clin. Cancer Res..

[117] Soria G., Ben-Baruch A. (2008). The inflammatory chemokines CCL2 and CCL5 in breast cancer. Cancer Lett..

[118] Bateman M.E., Strong A.L., Gimble J.M., Bunnell B.A. (2018). Concise review: Using fat to fight disease: A systematic review of nonhomologous adipose-derived stromal/stem cell therapies. Stem Cells.

[119] Nicolay N.H., Lopez Perez R., Debus J., Huber P.E. (2015). Mesenchymal stem cells—A new hope for radiotherapy-induced tissue damage?. Cancer Lett..

[120] Gronhoj C., Jensen D.H., Vester-Glowinski P., Jensen S.B., Bardow A., Oliveri R.S., Fog L.M., Specht L., Thomsen C., Darkner S. (2018). Safety and efficacy of mesenchymal stem cells for radiation-induced xerostomia: A randomized, placebo-controlled phase 1/2 trial (MESRIX). Int. J. Radiat. Oncol. Biol. Phys..

[121] Dufrane D., Docquier P.L., Delloye C., Poirel H.A., Andre W., Aouassar N. (2015). Scaffold-free three-dimensional graft from autologous adipose-derived stem cells for large bone defect reconstruction: clinical proof of concept. Medicine.

[122] Veriter S., Andre W., Aouassar N., Poirel H.A., Lafosse A., Docquier P.L., Dufrane D. (2015). human adipose-derived mesenchymal stem cells in cell therapy: Safety and feasibility in different “hospital exemption” clinical applications. PLoS ONE.

[123] Furia S., Cadenelli P., Andriani F., Scanagatta P., Duranti L., Spano A., Galeone C., Porcu L., Pastorino U. (2017). Autologous fat tissue grafting improves pulmonary healing after laser metastasectomy. Eur. J. Surg. Oncol..

[124] Bonomi A., Cocce V., Cavicchini L., Sisto F., Dossena M., Balzarini P., Portolani N., Ciusani E., Parati E., Alessandri G. (2013). Adipose tissue-derived stromal cells primed in vitro with paclitaxel acquire anti-tumor activity. Int. J. Immunopathol. Pharmacol..

[125] Paino F., la Noce M., di Nucci D., Nicoletti G.F., Salzillo R., de Rosa A., Ferraro G.A., Papaccio G., Desiderio V., Tirino V. (2017). Human adipose stem cell differentiation is highly affected by cancer cells both in vitro and in vivo: Implication for autologous fat grafting. Cell Death Dis..

[126] Papaccio F., Paino F., Regad T., Papaccio G., Desiderio V., Tirino V. (2017). Concise review: Cancer cells, cancer stem cells, and mesenchymal stem cells: Influence in cancer development. Stem Cells Transl Med..

[127] Gutowski K.A. (2009). Current applications and safety of autologous fat grafts: A report of the ASPS fat graft task force. Plast. Reconstr. Surg..

[128] Eaves F.F., Haeck P.C., Rohrich R.J. (2012). ASAPS/ASPS Position statement on stem cells and fat grafting. Plast. Reconstr. Surg..

[129] Brown S.A., Levi B., Lequeux C., Wong V.W., Mojallal A., Longaker M.T. (2010). Basic science review on adipose tissue for clinicians. Plast. Reconstr. Surg..

[130] de Francesco F., Ricci G., D’Andrea F., Nicoletti G.F., Ferraro G.A. (2015). Human adipose stem cells: From bench to bedside. Tissue Eng. Part B Rev..

[131] Zuk P.A., Zhu M., Mizuno H., Huang J., Futrell J.W., Katz A.J., Benhaim P., Lorenz H.P., Hedrick M.H. (2001). Multilineage cells from human adipose tissue: Implications for cell-based therapies. Tissue Eng..

[132] Strem B.M., Hicok K.C., Zhu M., Wulur I., Alfonso Z., Schreiber R.E., Fraser J.K., Hedrick M.H. (2005). Multipotential differentiation of adipose tissue-derived stem cells. Keio J. Med..

[133] Clarke D. (2018). Addressing particulates, extractables and leachables and the quality of single-use systems for cell and gene therapy manufacturing. Cell Gene Ther. Insights.

[134] Gentile P., Garcovich S. (2019). Concise review: Adipose-derived stem cells (ASCs) and adipocyte-secreted exosomal microRNA (A-SE-miR) modulate cancer growth and proMote wound repair. J. Clin. Med..

[135] Gentile P., Casella D., Palma E., Calabrese C. (2019). Engineered fat graft enhanced with adipose-derived stromal vascular fraction cells for regenerative medicine: Clinical, histological and instrumental evaluation in breast reconstruction. J. Clin. Med..

[136] Gentile P., Piccinno M.S., Calabrese C. (2019). Characteristics and potentiality of human adipose-derived stem cells (hASCs) obtained from enzymatic digestion of fat graft. Cells.

[137] Gentile P., de Angelis B., di Pietro V., Amorosi V., Scioli M.G., Orlandi A., Cervelli V. (2018). Gentle is better: The original “gentle technique” for fat placement in breast lipofilling. J. Cutan. Aesthet. Surg..

[138] Gentile P., Cervelli V. (2018). Adipose-derived stromal vascular fraction cells and platelet-rich plasma: Basic and clinical implications for tissue engineering therapies in regenerative surgery. Methods Mol. Biol..

[139] Fiaschetti V., Pistolese C.A., Fornari M., Liberto V., Cama V., Gentile P., Floris M., Floris R., Cervelli V., Simonetti G. (2013). Magnetic resonance imaging and ultrasound evaluation after breast autologous fat grafting combined with platelet-rich plasma. Plast. Reconstr. Surg..

[140] Gentile P., di Pasquali C., Bocchini I., Floris M., Eleonora T., Fiaschetti V., Floris R., Cervelli V. (2013). Breast reconstruction with autologous fat graft mixed with platelet-rich plasma. Surg Innov..

[141] Gentile P., Sarlo F., de Angelis B., de Lorenzo A., Cervelli V. (2016). Obesity phenotypes and resorption percentage after breast autologous fat grafting: Rule of low-grade inflammation. Adv. Biomed. Res..

[142] Bielli A., Scioli M.G., Gentile P., Cervelli V., Orlandi A. (2015). Adipose tissue-derived stem cell therapy for post-surgical breast reconstruction—More light than shadows. Adv. Clin. Exp. Med..

[143] Gentile P., Scioli M.G., Orlandi A., Cervelli V. (2015). Breast reconstruction with enhanced stromal vascular fraction fat grafting: What is the best method?. Plast. Reconstr. Surg. Glob. Open.

[144] Bielli A., Scioli M.G., Gentile P., Agostinelli S., Tarquini C., Cervelli V., Orlandi A. (2014). Adult adipose-derived stem cells and breast cancer: A controversial relationship. Springerplus.

[145] Gentile P., de Angelis B., Pasin M., Cervelli G., Curcio C.B., Floris M., di Pasquali C., Bocchini I., Balzani A., Nicoli F. (2014). Adipose-derived stromal vascular fraction cells and platelet-rich plasma: Basic and clinical evaluation for cell-based therapies in patients with scars on the face. J. Craniofac. Surg..

[146] Cervelli V., Bocchini I., di Pasquali C., de Angelis B., Cervelli G., Curcio C.B., Orlandi A., Scioli M.G., Tati E., Delogu P. (2013). platelet rich lipotransfert: Our experience and current state of art in the combined use of fat and PRP. Biomed. Res. Int..

[147] Gentile P., Scioli M.G., Bielli A., Orlandi A., Cervelli V. (2017). Comparing different nanofat procedures on scars: Role of the stromal vascular fraction and its clinical implications. Regen. Med..

[148] Gentile P., Scioli M.G., Bielli A., Orlandi A., Cervelli V. (2017). Concise review: The use of adipose-derived stromal vascular fraction cells and platelet rich plasma in regenerative plastic surgery. Stem Cells.

[149] Scioli M.G., Bielli A., Gentile P., Cervelli V., Orlandi A. (2017). Combined treatment with platelet-rich plasma and insulin favours chondrogenic and osteogenic differentiation of human adipose-derived stem cells in three-dimensional collagen scaffolds. J. Tissue Eng. Regen. Med..

[150] Scioli M.G., Cervelli V., Arcuri G., Gentile P., Doldo E., Bielli A., Bonanno E., Orlandi A. (2014). High insulin-induced down-regulation of Erk-1/IGF-1R/FGFR-1 signaling is required for oxidative stress-mediated apoptosis of adipose-derived stem cells. J. Cell Physiol..

[151] Scioli M.G., Bielli A., Gentile P., Mazzaglia D., Cervelli V., Orlandi A. (2014). The biomolecular basis of adipogenic differentiation of adipose-derived stem cells. Int. J. Mol. Sci..

[152] Gentile P., Orlandi A., Scioli M.G., di Pasquali C., Bocchini I., Cervelli V. (2012). Concise review: Adipose-derived stromal vascular fraction cells and platelet-rich plasma: Basic and clinical implications for tissue engineering therapies in regenerative surgery. Stem Cells Transl. Med..

[153] Cervelli V., Scioli M.G., Gentile P., Doldo E., Bonanno E., Spagnoli L.G., Orlandi A. (2012). Platelet-rich plasma greatly potentiates insulin-induced adipogenic differentiation of human adipose-derived stem cells through a serine/threonine kinase Akt-dependent mechanism and promotes clinical fat graft maintenance. Stem Cells Transl. Med..

[154] Cervelli V., Gentile P., de Angelis B., Calabrese C., di Stefani A., Scioli M.G., Curcio B.C., Felici M., Orlandi A. (2011). Application of enhanced stromal vascular fraction and fat grafting mixed with PRP in post-traumatic lower extremity ulcers. Stem Cell Res..

[155] Cervelli V., Lucarini L., Spallone D., Palla L., Colicchia G.M., Gentile P., de Angelis B. (2011). Use of platelet-rich plasma and hyaluronic acid in the loss of substance with bone exposure. Adv. Skin Wound Care.

[156] Gentile P., Bottini D.J., Spallone D., Curcio B.C., Cervelli V. (2010). Application of platelet-rich plasma in maxillofacial surgery: Clinical evaluation. J. Craniofac. Surg..

[157] Cervelli V., Gentile P., Scioli M.G., Grimaldi M., Casciani C.U., Spagnoli L.G., Orlandi A. (2009). Application of platelet-rich plasma in plastic surgery: Clinical and in vitro evaluation. Tissue Eng. Part C Methods.

[158] Gentile P., Cole J.P., Cole M.A., Garcovich S., Bielli A., Scioli M.G., Orlandi A., Insalaco C., Cervelli V. (2017). Evaluation of not-activated and activated PRP in hair loss treatment: Role of growth factor and cytokine concentrations obtained by different collection systems. Int. J. Mol. Sci..

[159] Gentile P., Garcovich S., Bielli A., Scioli M.G., Orlandi A., Cervelli V. (2015). The effect of platelet-rich plasma in hair regrowth: A randomized placebo-controlled trial. Stem Cells Transl. Med..

[160] Cole J.P., Cole M.A., Insalaco C., Cervelli V., Gentile P. (2017). Alopecia and platelet-derived therapies. Stem Cell Investig..

[161] Gentile P., Garcovich S., Scioli M.G., Bielli A., Orlandi A., Cervelli V. (2018). Mechanical and controlled PRP injections in patients affected by androgenetic alopecia. J. Vis. Exp..

[162] Gentile P., Colicchia G.M., Nicoli F., Cervelli G., Curcio C.B., Brinci L., Cervelli V. (2013). Complex abdominal wall repair using a porcine dermal matrix. Surg. Innov..

[163] Gentile P. (2019). Autologous cellular method using micrografts of human adipose tissue derived follicle stem cells in androgenic alopecia. Int. J. Mol. Sci..

[164] Gentile P., Garcovich S. (2019). Advances in regenerative stem cell therapy in androgenic alopecia and hair loss: Wnt pathway, Growth-factor, and mesenchymal stem cell signaling impact analysis on cell growth and hair follicle development. Cells.

[165] Gentile P., Scioli M.G., Bielli A., de Angelis B., de Sio C., de Fazio D., Ceccarelli G., Trivisonno A., Orlandi A., Cervelli V. (2019). Platelet-rich plasma and micrografts enriched with autologous human follicle mesenchymal stem cells improve hair re-growth in androgenetic alopecia. Biomolecular pathway analysis and clinical evaluation. Biomedicines.

[166] Gentile P., Scioli M.G., Bielli A., Orlandi A., Cervelli V. (2017). Stem cells from human hair follicles: First mechanical isolation for immediate autologous clinical use in androgenetic alopecia and hair loss. Stem Cell Investig..

[167] Cervelli V., Bottini D.J., Arpino A., Grimaldi M., Rogliani M., Gentile P. (2008). Bone-anchored implant in cosmetic finger reconstruction. Annales de Chirurgie Plastique Esthetique.

[168] Gentile P., Bottini D.J., Colicchia G.M., Trimarco A., Cervelli V. (2008). Burns: Bone-anchored, extra-oral implantology. J. Burn Care Res..

[169] Gentile P., Bottini D.J., Gravante G., Nicoli F., Caruso R., Cervelli V. (2008). The use of bone-anchored implants for absent ear. J. Craniofac. Surg..

[170] Gentile P., Nicoli F., Caruso R., Gravante G., Cervelli V. (2009). Alternative strategy to reconstruct the nose after excision: Extra-oral implant anchored to bone. Br. J. Oral. Maxillofac. Surg..

[171] Cervelli V., Bottini D.J., Arpino A., Colicchia G.M., Mugnaini F., Trimarco A., Gentile P., Grimaldi M. (2006). Orbital reconstruction: Bone-anchored implants. J. Craniofac. Surg..

[172] Bottini D.J., Gentile P., Donfrancesco A., Fiumara L., Cervelli V. (2008). Augmentation rhinoplasty with autologous grafts. Aesthet. Plast Surg..

[173] Gentile P., Cervelli V. (2009). Nasal dorsum reconstruction with 11th rib cartilage and auricular cartilage grafts. Ann. Plast. Surg..

[174] Cervelli V., Bottini D.J., Gentile P., Fantozzi L., Arpino A., Cannatà C., Fiumara L., Casciani C.U. (2006). Reconstruction of the nasal dorsum with autologous rib cartilage. Ann. Plast. Surg..

[175] Pizzicannella J., Gugliandolo A., Orsini T., Fontana A., Ventrella A., Mazzon E., Bramanti P., Diomede F., Trubiani O. (2019). Engineered extracellular vesicles from human periodontal-ligament stem cells increase VEGF/VEGFR2 expression during bone regeneration. Front. Physiol..

[176] Mammana S., Gugliandolo A., Cavalli E., Diomede F., Iori R., Zappacosta R., Bramanti P., Conti P., Fontana A., Pizzicannella J. (2019). Human gingival mesenchymal stem cells pretreated with vesicular moringin nanostructures as a new therapeutic approach in a mouse model of spinal cord injury. J. Tissue Eng. Regen. Med..

[177] Trubiani O., Ballerini P., Murmura G., Pizzicannella J., Giuliani P., Buccella S., Caputi S. (2012). Toll-like receptor 4 expression, interleukin-6, -8 and Ccl-20 release, and NF-KB translocation in human periodontal ligament mesenchymal stem cells stimulated with LPS-P. Gingivalis Eur. J. Inflamm..

[178] Campagnoli C. (2001). Identification of mesenchymal stem/progenitor cells in human first-trimester fetal blood, liver, and bone marrow. Blood.

[179] Baksh D., Davies J.E., Zandstra P.W. (2003). Adult human bone marrow–derived mesenchymal progenitor cells are capable of adhesion-independent survival and expansion. Exp. Hematol..

[180] Muschler G.F., Nitto H., Boehm C.A., Easley K.A. (2001). Age-and gender-related changes in the cellularity of human bone marrow and the prevalence of osteoblastic progenitors. J. Orthop. Res..

[181] Muschler G.F., Boehm C., Easley K. (1997). Aspiration to obtain osteoblast progenitor cells from human bone marrow: The influence of aspiration volume. J. Bone Jt. Surg. Am..

[182] Peng L., Jia Z., Yin X., Zhang X., Liu Y., Chen P., Ma K., Zhou C. (2008). Comparative analysis of mesenchymal stem cells from bone marrow, cartilage, and adipose tissue. Stem Cells Dev..

[183] Kern S., Eichler H., Stoeve J., Klüter H., Bieback K. (2006). Comparative analysis of mesenchymal stem cells from bone marrow, umbilical cord blood, or adipose tissue. Stem Cells.

[184] Yoshimura H., Muneta T., Nimura A., Yokoyama A., Koga H., Sekiya I. (2007). Comparison of rat mesenchymal stem cells derived from bone marrow, synovium, periosteum, adipose tissue, and muscle. Cell Tissue Res..

[185] Rebelatto C.K., Aguiar A.M., Moretão M.P., Senegaglia A.C., Hansen P., Barchiki F., Oliveira J., Martins J., Kuligovski C., Mansur F. (2008). Dissimilar differentiation of mesenchymal stem cells from bone marrow, umbilical cord blood, and adipose tissue. Exp. Biol. Med..

[186] Oedayrajsingh-Varma M., van Ham S., Knippenberg M., Helder M., Klein-Nulend J., Schouten T., Ritt M., van Milligen F. (2006). Adipose tissue–derived mesenchymal stem cell yield and growth characteristics are affected by the tissue-harvesting procedure. Cytotherapy.

[187] Rad M.R., Bohloli M., Rahnama M.A., Anbarlou A., Nazeman P., Khojasteh A. (2017). Impact of tissue harvesting sites on the cellular behaviors of adipose-derived stem cells: Implication for bone tissue engineering. Stem Cells Int..

[188] Ardeshirylajimi A., Rafeie F., Zandi-Karimi A., Jaffarabadi G.A., Mohammadi-Sangcheshmeh A., Samiei R., Toghdory A., Seyedjafari E., Hashemi S.M., Cinar M.U. (2016). Fat harvesting site is an important determinant of proliferation and pluripotency of adipose-derived stem cells. Biologicals.

